# To Correspondents and Publishers

**Published:** 1845-04

**Authors:** 


### THE MEDICAL EXAMINER.

PHILADELPHIA, APRIL, 1845.

*To Correspondents and Publishers.*—The Editor regrets that he has been unable to notice all the favours sent to him for the present number. The press has literally teemed with medical books of late, and as a consequence, our account of them has necessarily been brief, and sometimes delayed longer than we could wish.

